# Indomethacin Enhances Type 1 Cannabinoid Receptor Signaling

**DOI:** 10.3389/fnmol.2019.00257

**Published:** 2019-10-18

**Authors:** Robert B. Laprairie, Kawthar A. Mohamed, Ayat Zagzoog, Melanie E. M. Kelly, Lesley A. Stevenson, Roger Pertwee, Eileen M. Denovan-Wright, Ganesh A. Thakur

**Affiliations:** ^1^College of Pharmacy and Nutrition, University of Saskatchewan, Saskatoon, SK, Canada; ^2^Department of Pharmacology, Dalhousie University, Halifax, NS, Canada; ^3^Department of Ophthalmology and Visual Sciences, Dalhousie University, Halifax, NS, Canada; ^4^School of Medical Sciences, The Institute of Medical Sciences, University of Aberdeen, Aberdeen, United Kingdom; ^5^Center for Drug Discovery, Department of Pharmaceutical Sciences, School of Pharmacy, Bouvé College of Health Sciences, Northeastern University, Boston, MA, United States

**Keywords:** cannabinoid, indomethacin, cannabinoid receptor, allosteric modulator, molecular pharmacology, cell signaling

## Abstract

In addition to its known actions as a non-selective cyclooxygenase (COX) 1 and 2 inhibitor, we hypothesized that indomethacin can act as an allosteric modulator of the type 1 cannabinoid receptor (CB1R) because of its shared structural features with the known allosteric modulators of CB1R. Indomethacin enhanced the binding of [^3^H]CP55940 to hCB1R and enhanced AEA-dependent [^35^S]GTPγS binding to hCB1R in Chinese hamster ovary (CHO) cell membranes. Indomethacin (1 μM) also enhanced CP55940-dependent βarrestin1 recruitment, cAMP inhibition, ERK1/2 and PLCβ3 phosphorylation in HEK293A cells expressing hCB1R, but not in cells expressing hCB2R. Finally, indomethacin enhanced the magnitude and duration of CP55940-induced hypolocomotion, immobility, hypothermia, and anti-nociception in C57BL/6J mice. Together, these data support the hypothesis that indomethacin acted as a positive allosteric modulator of hCB1R. The identification of structural and functional features shared amongst allosteric modulators of CB1R may lead to the development of novel compounds designed for greater CB1R or COX selectivity *and* compounds designed to modulate both the prostaglandin and endocannabinoid systems.

## Introduction

The endocannabinoid system consists of endogenous cannabinoids such as anandamide (AEA) and 2-arachidonoylglycerol (2-AG), their anabolic and catabolic enzymes, and receptors including the type 1 and 2 cannabinoid receptors (CB1R, CB2R). There is a growing interest in defining the actions of drugs that modulate the activity of the endocannabinoid system. Specifically, compounds that selectively enhance the activity of CB1R may be used in the treatment of pain, depression, and neurodegenerative diseases (Ross, 2007). Compounds that directly activate CB1R – orthosteric agonists – have limited potential as novel therapeutic compounds because of their psychoactivity (Ross, 2007; Pertwee, 2008). Positive allosteric modulators (PAM) of CB1R bind to a CB1R site different from the CB1R site targeted by endocannabinoids and enhance the binding of orthosteric ligands to CB1R, and/or enhance orthosteric ligand-dependent signaling without intrinsic efficacy (Ross, 2007). CB1R PAMs are being developed as novel therapeutic compounds for a wide range of disease states (Price et al., 2005; Ahn et al., 2012; Pamplona et al., 2012).

Existing allosteric modulators of CB1R include Org27569, PSNCBAM-1, lipoxin A_4_, ZCZ011, cannabidiol (CBD), and GAT211 (Price et al., 2005; Ahn et al., 2012; Pamplona et al., 2012; Ignatowska-Jankowska et al., 2015; Laprairie et al., 2015, 2017; Tham et al., 2018). Org27569 and PSNCBAM-1 both enhance orthosteric ligand binding to CB1R, but diminish CB1R-dependent ERK1/2 phosphorylation and βarrestin recruitment (Price et al., 2005; Ahn et al., 2012; Cawston et al., 2013; Shore et al., 2014). Org27569 and PSNCBAM-1 also display inverse agonist activity at cAMP and ERK1/2 pathways in the absence of orthosteric ligands, indicating these compounds are not pure allosteric modulators (Ahn et al., 2012; Shore et al., 2014). Lipoxin A_4_ is a PAM of ligand binding and orthosteric agonist-dependent cAMP inhibition at CB1R, but this compound is unstable and displays low potency (high micromolar) *in vitro*, limiting its therapeutic utility (Pamplona et al., 2012). CBD is a negative allosteric modulator (NAM) of CB1R-dependent ERK1/2 and PLCβ3 phosphorylation, βarrestin recruitment, and cAMP inhibition that reduces CP55940 binding at concentrations >1 μM (Laprairie et al., 2019). ZCZ011 and GAT211 are both potent and efficacious CB1R PAMs; these lead compounds are being used as scaffolds for the development of more specific, potent, and efficacious CB1R PAMs (Ignatowska-Jankowska et al., 2015; Laprairie et al., 2017, 2019).

**FIGURE 1:**
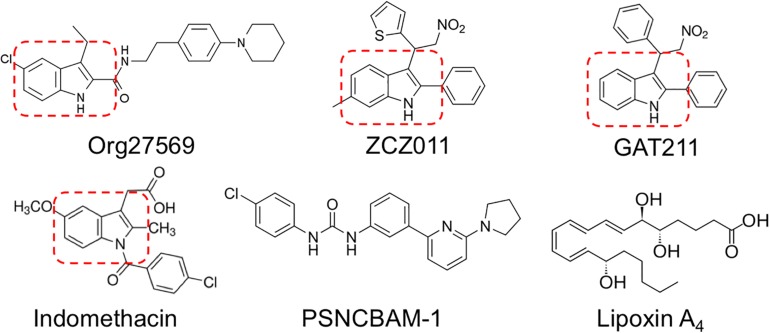
Previously described allosteric modulators of CB1R.

Org27569, ZCZ011, and GAT211 share in common a 2- and 3-alkyl-group-substituted indole ring (indole-2-carboxamides) (Price et al., 2005; Ahn et al., 2012; Cawston et al., 2015; Ignatowska-Jankowska et al., 2015; Laprairie et al., 2017), suggesting this is an important structural requirement for allosteric modulators of CB1R (reviewed in Lu et al., 2018) (Figure 1). CB1R allosteric modulator activity is maintained or improved by C-5 substitution of Org27569 and GAT211 (Cawston et al., 2015; Hurst et al., 2019). PSNCBAM-1 and lipoxin A_4_ do not contain substituted indole rings; however, both contain structural features that mimic the space and charge occupied by an indole ring (Ahn et al., 2012; Pamplona et al., 2012). Further, Cawston et al. (2015) recently demonstrated that varying the substituents around indole-2-carboxamides can affect the temporal activity of Org27569 derivatives, without affecting the NAM activity these compounds have on CB1R-mediated signaling. Based on the presence of an indole-2-carboxamide, and literature demonstrating the potential actions that might indicate an undocumented CB1R allosteric modulatory activity (Cawston et al., 2015; Lu et al., 2018), we identified indomethacin as a potential allosteric modulator of CB1R.

The non-steroidal anti-inflammatory drug (NSAID) indomethacin acts as high-affinity non-selective cyclooxygenase 1 and 2 (COX-1, COX-2) inhibitor, fatty acid amide hydrolase (FAAH) inhibitor, prostaglandin receptor 2 agonist, and β_2_ andrenoreceptor antagonist (Fowler et al., 1997a). The substituted indole ring of indomethacin is unique among NSAIDs (Fowler et al., 1997a). Indomethacin has been shown to enhance AEA- and CB1R-dependent signaling *in vivo*, but these effects were independent of direct CB1R agonism or an increase in AEA levels (Wiley et al., 2006; Parvathy and Masocha, 2015). Indomethacin, unlike other NSAIDs, produces several neurologic side effects, including vertigo, dizziness, blurred vision, and psychosis, that may be the result of the endocannabinoid system and/or CB1R modulation (Fowler, 1987).

### Objective of This Study

Based on the structural similarities of indomethacin to known CB1R allosteric modulators, and the neurologic effects associated with indomethacin use, the objective of this study was to determine whether indomethacin acted as an allosteric modulator of CB1R. To accomplish this objective, indomethacin’s *in vitro* effects on orthosteric ligand binding to CB1R, G protein-coupling to CB1R, and CB1R-mediated signal transduction; and *in vivo* effects on CP55940-dependent anti-nociception, catalepsy, hypothermia, and locomotion were determined.

## Materials and Methods

### Compounds

CP55940 [(-)-*cis*-3-[2-Hydroxy-4-(1,1-dimethylheptyl)phenyl]-*trans*-4-(3-hydroxypropyl)cyclohexanol] was purchased from Tocris Bioscience (Bristol, United Kingdom). AEA and indomethacin were purchased from Sigma-Aldrich (Poole, Dorset, United Kingdom). [^3^H]CP55940 (174.6 Ci/mmol) and [^35^S]GTPγS (1250 Ci/mmol) were obtained from PerkinElmer (Seer Green, Buckinghamshire, United Kingdom), GTPγS from Roche Diagnostic (Burgess Hill, West Sussex, United Kingdom), and GDP from Sigma-Aldrich. Compounds were dissolved in DMSO (final concentration of 0.1% in assay media for all assays) and added directly to the media at the concentrations and times indicated.

### Cell Culture

Chinese hamster ovary (CHO) cells transfected with cDNA encoding human cannabinoid CB1R or CB2R were maintained at 37°C, 5% CO_2_ in DMEM F-12 HAM, supplemented with 1 mM L-glutamine, 10% FBS, and 0.6% Pen/Strep for all cells, together with hygromycin B (300 mg/ml) and G418 (600 mg/ml) for the human CB1R CHO cells or with G418 (400 mg/ml) for the human CB2R CHO cells (Bolognini et al., 2010). For membrane preparation, cells were removed from flasks by scraping, centrifuged, and then frozen as a pellet at −20°C until required. Before use in a radioligand binding assay, cells were defrosted, diluted in Tris buffer (50 mM Tris–HCl and 50 mM Tris–base) and homogenized with a 1 mL hand-held homogenizer (Bolognini et al., 2010).

HitHunter (cAMP) and PathHunter (βarrestin2) CHO-K1 cells stably expressing human CB1R (hCB1R) from DiscoveRx^®^ (Eurofins, Fremont, CA, United States) were maintained at 37°C, 5% CO_2_ in F-12 DMEM containing 10% FBS and 1% penicillin-streptomycin with 800 μg/mL geneticin (HitHunter) or 800 μg/mL geneticin and 300 μg/mL hygromycin B (PathHunter).

Human embryonic kidney (HEK) 293A cells were from the American Type Culture Collection (ATCC, Manassas, VA, United States). HEK293A cells were maintained at 37°C, 5% CO_2_ in DMEM supplemented with 10% FBS and 1% Pen/Strep.

HEK293A Cignal Lenti CRE (HEK-CRE) reporter cells were provided by Dr. Christopher J. Sinal (Dalhousie University, Halifax, NS, Canada). The HEK-CRE cells stably express the firefly luciferase gene driven by tandem repeat elements of the cAMP transcriptional response element (Qiagen, Toronto, ON, Canada). Thus, luciferase activity is directly proportional to the level cAMP/PKA pathway activation or inhibition. HEK-CRE cells were maintained at 37°C, 5% CO_2_ in DMEM supplemented with 10% FBS, 1% Pen/Strep, and 200 μg/mL puromycin.

### CHO Cell Membrane Preparations

CHO cells stably expressing hCB1R or hCB2R were disrupted by cavitation in a pressure cell and membranes were sedimented by ultracentrifugation, as described previously (Bolognini et al., 2012). The pellet was resuspended in TME buffer (50 mM Tris–HCl, 5 mM MgCl_2_, 1 mM EDTA, pH 7.4) and membrane proteins were quantified with a Bradford dye-binding method (Bio-Rad Laboratories).

### Radioligand Displacement Assays

Assays were carried out with [^3^H]CP55940 and Tris binding buffer (50 mM Tris–HCl, 50 mM Tris–base, 0.1% BSA, pH 7.4), total assay volume 500 μL, using the filtration procedure described previously by Ross et al. (1999) and Baillie et al. (2013). Binding was initiated by the addition of transfected human CB1R or CB2R CHO cell membranes (50 μg protein per well). All assays were performed at 37°C for 60 min before termination by the addition of ice-cold Tris binding buffer, followed by vacuum filtration using a 24-well sampling manifold (Brandel Cell Harvester; Brandel Inc., Gaithersburg, MD, United States) and Brandel GF/B filters that had been soaked in wash buffer at 4°C for at least 24 h. Each reaction well was washed six times with a 1.2 mL aliquot of Tris binding buffer. The filters were oven-dried for 60 min and then placed in 3 ml of scintillation fluid (Ultima Gold XR, PerkinElmer, Seer Green, Buckinghamshire, United Kingdom). Radioactivity was quantified by liquid scintillation spectrometry. Specific binding was defined as the difference between the binding that occurred in the presence and absence of 1 μM unlabeled CP55940. The concentration of [^3^H]CP55940 used in our displacement assays was 0.7 nM. Indomethacin was stored as stock solutions of 10 mM in DMSO, the vehicle concentration in all assay wells was 0.1% DMSO.

### Dissociation Binding Assay

Membranes obtained from CHO cells transfected with hCB1R were incubated at 24°C in a 96 deep-well block immersed in a water bath (50 μg protein per well), together with 350 μL of assay buffer (50 mM Tris HCl, 50 mM Tris Base and 0.1% w/v BSA, pH 7.4), and 50 μL [^3^H]CP55940 (7 nM) in each well for 60 min to allow full association of [^3^H]CP55940 to occur. Dissociation of [^3^H]CP55940 was monitored at various times over a further period of 60 min after the addition of 1 μM unlabeled CP55940 in the presence or absence of 1 μM indomethacin at 24°C. The assay was terminated by rapid filtration onto GF/B filters pre-soaked in assay buffer using a Brandel cell harvester. The filters were washed six times with the ice-cold buffer before being dried in a heated cabinet. Filters were placed in vials to which 3 mL Ultima Gold scintillation fluid was added. The radioactivity in each vial was then counted for 3 min in a Tri-Carb liquid scintillation counter.

### [^35^S]GTPγS Binding Assay

Human CB1R and CB2R CHO cell membranes (25 μg protein) were preincubated for 30 min at 30°C with adenosine deaminase (0.5 IU/ml). The membranes were then incubated with the agonist ± indomethacin or vehicle for 60 min at 30°C in assay buffer (50 mM Tris–HCl; 50 mM Tris–Base; 5 mM MgCl_2_; 1 mM EDTA; 100 mM NaCl; 1 mM DTT; 0.1% BSA) in the presence of 0.1 nM [^35^S]GTPγS and 30 μM GDP, in a final volume of 500 μL. Binding was initiated by the addition of [^35^S]GTPγS. Non-specific binding was measured in the presence of 30 μM GTPγS. The reaction was terminated by rapid vacuum filtration (50 mM Tris–HCl; 50 mM Tris–Base; 0.1% BSA) using a 24-well sampling manifold (cell harvester; Brandel, Gaithersburg, MD, United States) and GF/B filters (Whatman, Maidstone, United Kingdom) that had been soaked in buffer (50 mM Tris–HCl; 50 mM Tris–Base; 0.1% BSA) for at least 24 h. Each reaction tube was washed six times with a 1.2-mL aliquot of ice-cold wash buffer. The filters were oven-dried for at least 60 min and then placed in 3 mL of scintillation fluid (Ultima Gold XR, PerkinElmer, Cambridge, United Kingdom). Radioactivity was quantified by liquid scintillation spectrometry.

### RT-PCR

RNA was harvested from HEK293A cells using the Trizol^®^ (Invitrogen, Burlington, ON, Canada) extraction method according to the manufacturer’s instruction. Reverse transcription reactions were carried out with SuperScript III^®^ reverse transcriptase (+RT; Invitrogen), or without (−RT) as a negative control for use in subsequent PCR experiments according to the manufacturer’s instructions. Two micrograms of RNA were used per RT reaction for cDNA synthesis. PCR reactions were composed of 1X *Taq* polymerase PCR buffer, a primer-specific concentration of MgCl_2_ (Supplementary Table S1), 0.3 mM dNTPs, 0.5 μM each of forward and reverse primers (Supplementary Table S1), 1 μL cDNA, and 1.25 U *Taq* polymerase, to a final volume of 20 μL with dH_2_O (Fermentas). The PCR program was: 95°C for 10 min, 35 cycles of 95°C 30 s, a primer-specific annealing temperature (Supplementary Table S1) for 30 min, and 72°C for 1 min.

### Plasmids

Human CB1R- and CB2R-green fluorescent protein^2^ (GFP^2^) C-terminal fusion protein was generated using the pGFP^2^-N3 (PerkinElmer, Waltham, MA, United States) plasmid, as described previously (Bagher et al., 2013). Human βarrestin1-*Renilla* luciferase II (RlucII) C-terminal fusion protein was generated using the pcDNA3.1 plasmid and provided by Dr. Denis J. Dupré (Dalhousie University, Halifax, NS, Canada). The GFP^2^-Rluc fusion construct, and Rluc plasmids have also been described (Bagher et al., 2013).

### Bioluminescence Resonance Energy Transfer^2^

Direct interactions between CB1R or CB2R and βarrestin1 were quantified via Bioluminescence Resonance Energy Transfer^2^ (BRET^2^) (James et al., 2006). Cells were transfected with the indicated GFP^2^ and *Rluc* constructs using Lipofectamine 2000, according to the manufacturer’s instructions (Invitrogen) and treated as previously described (Laprairie et al., 2014). Briefly, 48 h post-transfection cells were washed twice with cold PBS and suspended in BRET buffer [PBS supplemented with glucose (1 mg/mL), benzamidine (10 mg/mL), leupeptin (5 mg/mL), and a trypsin inhibitor (5 mg/mL)]. Cells were treated with compounds as indicated (PerkinElmer) and coelenterazine 400a substrate (50 μM; Biotium, Hayward, CA, United States) was added. Light emissions were measured at 460 nm (Rluc) and 510 nm (GFP^2^) using a Luminoskan Ascent plate reader (Thermo Scientific, Waltham, MA, United States), with an integration time of 10 s and a photomultiplier tube voltage of 1200 V. BRET efficiency (BRET_Eff_) was determined using previously described methods (Bagher et al., 2013; Laprairie et al., 2014). Data are presented as % of the maximal response to CP55940.

### In-Cell Westerns

Cells were fixed for 10 min at room temperature with 4% paraformaldehyde and washed three times with 0.1 M PBS for 5 min each. Cells were incubated with blocking solution (PBS, 20% Odyssey blocking buffer, and 0.1% TritonX-100) for 1 h at room temperature. Cells were incubated with primary antibody solutions directed against pERK1/2(Y205/185), ERK1/2, pPLCβ3(S573), or PLCβ3 (Santa Cruz Biotechnology) diluted (1:200) in blocking solution overnight at 4°C. Cells were washed three times with PBS for 5 min each. Cells were incubated in IR^CW700dye^ or IR^CW800dye^ (1:500; Rockland Immunochemicals) and washed three times with PBS for 5 min each. Analyses were conducted using the Odyssey Imaging system and software (version 3.0; Li-Cor). Data are presented as % of the maximal response to CP55940.

### cAMP Luciferase Reporter Assay

HEK-CRE cells were transfected with CB1R-GFP^2^ or CB2R-GFP^2^. Forty-eight hours post-transfection cells were washed twice with cold PBS and suspended in BRET buffer. Cells were dispensed into 96-well plates (10,000 cells/well) and treated with 10 μM forskolin and ligands (PerkinElmer). Media was aspirated from cells and cells were lysed with passive lysis buffer for 20 min at room temperature (Promega, Oakville, ON, Canada). Twenty microliters of cell lysate were mixed with luciferase assay reagent (50 μM; Promega, Oakville, ON, Canada) and light emissions were measured at 405 nm using a Luminoskan Ascent plate reader (Thermo Scientific, Waltham, MA, United States), with an integration time of 10 s and a photomultiplier tube voltage of 1200 V. Data are presented as % inhibition of forskolin response.

### HitHunter cAMP Assay

Inhibition of forskolin-stimulated cAMP was determined using the DiscoveRx HitHunter assay in hCB1R CHO-K1 cells. Cells (20,000 cells/well in low-volume 96 well plates) were incubated overnight in Opti-MEM (Invitrogen) containing 1% FBS at 37°C and 5% CO_2_. Following this, Opti-MEM media was removed and replaced with cell assay buffer (DiscoveRx) and cells were co-treated at 37°C with 10 μM forskolin and ligands for 90 min. cAMP antibody solution and cAMP working detection solutions were then added to cells according to the manufacturer’s directions (DiscoveRx^®^) and cells were incubated for 60 min at room temperature. cAMP solution A was added according to the manufacturer’s directions (DiscoveRx^®^) and cells were incubated for an additional 60 min at room temperature before chemiluminescence was measured on a Cytation 5 plate reader (top read, gain 200, integration time 10,000 ms). Data are presented as % inhibition of forskolin response.

### PathHunter CB1R βarrestin2 Assay

βarrestin2 recruitment was determined using the hCB1R CHO-K1 cell PathHunter assay (DiscoveRx^®^). Cells (20,000 cells/well in low-volume 96 well plates) were incubated overnight in Opti-MEM (Invitrogen) containing 1% FBS at 37°C and 5% CO_2_. Following this, cells were co-treated at 37°C with ligands for 90 min. Detection solution was then added to cells according to the manufacturer’s directions (DiscoveRx^®^) and cells were incubated for 60 min at room temperature. Chemiluminescence was measured on a Cytation 5 plate reader (top read, gain 200, integration time 10,000 ms). Data are presented as % of the maximal response to CP55940.

### Animals and Tetrad Testing

Seven-week old, male, C57BL/6J mice (mean weight 25.2 ± 0.5 g) were purchased from The Jackson Laboratory (Bar Harbor, ME, United States). Animals were group housed (5 per cage) with *ad libitum* access to food, water, and environmental enrichment and maintained on a 12 h light/dark cycle. Mice were randomly assigned to receive 2 volume-matched *i.p.* injections of vehicle (10% DMSO in saline), 0.1 mg/kg CP55940 + vehicle, 2 mg/kg indomethacin + vehicle, 0.1 mg/kg CP55940 + 2 or 4 mg/kg indomethacin (*n* = 5 per group). All protocols were in accordance with the guidelines detailed by the Canadian Council on Animal Care (CCAC; Ottawa ON: Vol. 1, 2nd Ed., 1993; Vol. 2, 1984), approved by the Carleton Animal Care Committee at Dalhousie University. In keeping with the ARRIVE guidelines, power analyses were conducted to determine the minimum number of animals required for the study and animals were purchased – rather than bred – to limit animal waste, and all assessments of animal behavior were made by individuals blinded to treatment group (Kilkenny et al., 2010).

Anti-nociception was determined by assessing tail flick latency immediately prior to injection and 0.5, 1, and 4 h following injection. Mice were restrained with their tails placed ∼1 cm into water held at 52°C and the time until the tail was removed was recorded as tail flick latency (s). Observations were ended at 10 s.

Catalepsy was assessed in the ring holding assay immediately prior to injection and 1 and 4 h following injection. The mice were placed such that their forepaws clasped a 5 mm ring positioned 5 cm above the surface of the testing space. The length of time the ring was held was recorded (s). The trial was ended if the mouse turned its head or body, or made three consecutive escape attempts.

Internal body temperature was measured via rectal thermometer immediately prior to injection and 0.5, 1, and 4 h following injection.

Locomotion was assessed in the open field test immediately prior to injection and 1 and 4 h following injection. Mice were placed in an open space 90 cm × 60 cm and total distance was recorded for 5 min. Data are displayed as the total distance travelled over 5 min (m).

### Statistical Analyses

Data for [^3^H]CP55940 binding and [^35^S]GTPγS binding data are shown as % change from a basal level. In-cell westerns, BRET, and PathHunter data are shown as % of maximal CP55940 response. cAMP luciferase and HitHunter data are shown as % of forskolin response. Concentration-response curves (CRC) were fit using non-linear regression with variable slope (four parameters) and used to calculate EC_50_, *E*_min_, and *E*_max_ (GraphPad, Prism, v. 8.0). CRC were fit to the operational model of Black and Leff (1983) to calculate bias (ΔΔLogR) according to previously described methods and using CP55940 as the reference agonist (Laprairie et al., 2017). Statistical analyses were conducted by Student’s one

sample *t*-test, one- or two-way analysis of variance (ANOVA), as indicated in the figure legends, using GraphPad. *Post hoc* analyses were performed using Bonferroni’s (two-way ANOVA) or Tukey’s (one-way ANOVA) tests. Homogeneity of variance was confirmed using Bartlett’s test. All results are reported as the mean ± the standard error of the mean (SEM) or 95% confidence interval (CI), as indicated. *P*-values < 0.05 were considered to be significant.

### Receptor Modeling and Ligand Docking

The 2.8 Å agonist-bound (PDB ID: 5XRA) (Hua et al., 2017) human CB1R crystal structure was used. Amino acid position is indicated according to the Ballesteros and Weinstein method of residue numbering [i.e., single letter amino acid abbreviation, transmembrane helix number, the residue position relative to the most conserved position (e.g., F2.62)] (Ballesteros and Weinstein, 1995). Ligand “.mol2” structure and formula files for indomethacin were downloaded from ZINC (Irwin et al., 2012). Three-dimensional models of human CB1R were generated in Swiss-MODEL from the template structures (5XRA) (Arnold et al., 2006; Kiefer et al., 2009). All settings were kept at default. Ligands were docked to model receptors using AutoDock 4.2.6 (Morris et al., 2009) by Lamarckian genetic algorithm (Hurst et al., 2006). AutoDock uses a Monte Carlo simulated annealing algorithm to explore a defined grid within the virtual space of a protein model with a selected ligand. The ligand is used to probe the defined grid space via molecular affinity potentials in various conformations of ligand and receptor. The binding site of the models were defined using the AutoGrid program within AutoDock and the grid box was set to dimensions of 20 × 20 × 20 Å in order to include the entire extracellular surface and transmembrane regions of the model receptors. The rigidity parameters were set for the receptor and the ligands were kept flexible. All other parameters were set to default. The AutoDock algorithm AutoDock Vina 1.1.2 (Morris et al., 2009; Trott and Olson, 2010) was used to fit the ligand to the template. The best conformation for each ligand-receptor is based on the lowest binding energy among eight bioactive conformations generated by eight repeated program iterations.

## Results

### Radioligand Binding and [^35^S]GTPγS Binding Assay

**FIGURE 2:**
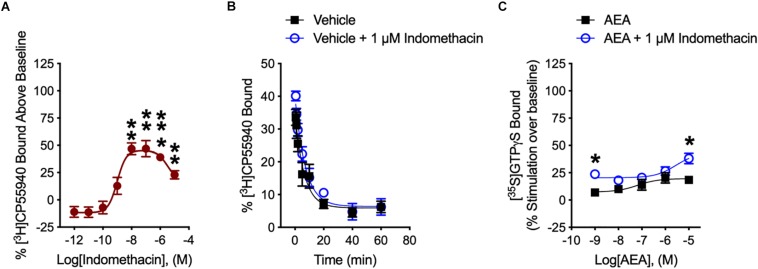
[^3^H]CP55940 and [^35^S]GTPγS binding to hCB1R. **(A)** [^3^H]CP55940 (0.7 nM) binding to membranes obtained from CHO cells transfected with hCB1R was measured in the presence of indomethacin. Symbols represent mean percentage changes in [^3^H]CP55940 binding values ± SEM. Asterisks indicate mean values that are significantly different from zero via Student’s one sample *t*-test (^∗∗^*P* < 0.01, ^∗∗∗^*P* < 0.001). Data are presented as mean values ± SEM. *N* = 4–6. **(B)** Effect of 1 μM indomethacin on the kinetics of [^3^H]CP55940 for its dissociation from binding sites on membranes obtained from hCB1R CHO cells. Data were best fitted using a one-phase dissociation model. Data are presented as mean values ± SEM. *N* = 5. **(C)** The effects of indomethacin on [^35^S]GTPγS binding in CHO cells expressing hCB1R treated with AEA in the presence of DMSO or 1 μM indomethacin. Asterisks indicate mean values that are significantly different from zero via Student’s one sample *t-*test (^∗^*P* < 0.05). Data are mean ± SEM. *N* = 5–6.

We determined how indomethacin modulated the binding of CP55940 – a high affinity, synthetic CB1R reference ligand – to hCB1R. Indomethacin enhanced [^3^H]CP55940 binding to hCB1R in CHO cell membranes between 10 nM and 10 μM (Figure 2A). The indomethacin concentration-[^3^H]CP55940 binding relationship was bell-shaped, with the greatest enhancement of binding occurring at 10 and 100 nM, suggesting that indomethacin may only enhance orthosteric ligand binding within a narrow concentration range, and at higher doses indomethacin may have reduced CP55940-hCB1R binding (Figure 2A). Indomethacin (1 μM) did not change the rate of dissociation of [^3^H]CP55940 compared to vehicle (Figure 2B and Table 1). Therefore, indomethacin enhanced the binding affinity of CP55940 at hCB1R, but did not change the dissociation rate of CP55940. Overall, these data are consistent with indomethacin acting as a PAM of orthosteric ligand binding at hCB1R. In order to assess the ability of indomethacin to modulate G protein activation, [^35^S]GTPγS binding assays were conducted in CHO cells stably expressing hCB1R. In the presence of 1 nM and 10 μM AEA, 1 μM indomethacin enhanced the [^35^S]GTPγS binding to hCB1R (Figure 2C). Indomethacin did not effect [^35^S]GTPγS binding to hCB2R (data not shown).

**TABLE 1:** Effect of indomethacin on the mean [^3^H]CP55940 of dissociation rate from membranes of CHO cells expressing hCB1R.

	***t*_1__/__2_ (min) (95% CI)^a^**
DMSO	4.75 (2.89–13.4)
+1 μM indomethacin	4.67 (3.17–8.80)

*^*a*^Data were best fitted using a one-phase dissociation model. *N* = 5.*

### βarrestin1, ERK1/2, PLCβ3, and cAMP

**FIGURE 3:**
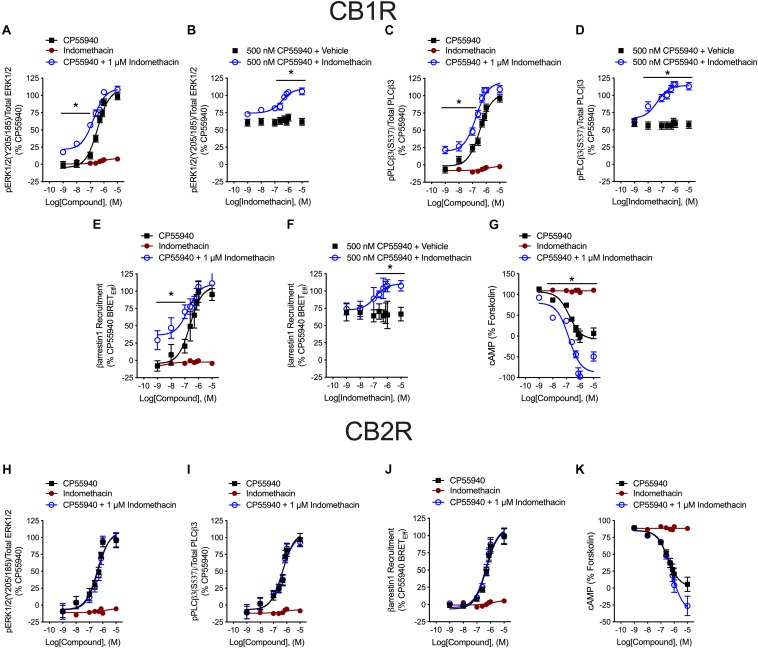
hCB1R and hCB2R signaling in the presence of indomethacin. **(A–D)** HEK293A cells expressing hCB1R-GFP^2^ were treated with 1 nM–10 μM CP55940 ± 1 μM indomethacin **(A,C)** or 1 nM–10 μM indomethacin ±500 nM CP55940 **(B,D)** for 10 min and ERK1/2 **(A,B)** or PLCβ3 **(C,D)** phosphorylation was measured. **(E,F)** HEK293A cells expressing hCB1R-GFP^2^ and βarrestin1-Rluc were treated with 1 nM–10 μM CP55940 ± 1 μM indomethacin **(E)** or 1 nM–10 μM indomethacin ±500 nM CP55940 **(F)** for 30 min and BRET^2^ was measured. **(G)** HEK-CRE cells expressing hCB1R-GFP^2^ were treated with 10 μM forskolin, 1 nM–10 μM CP55940 ± 1 μM indomethacin for 1 h. ^∗^*P* < 0.01 compared to CP55940 alone within dose as determined via one-way ANOVA followed by Tukey’s *post hoc* analysis. Data are mean ± SEM. *N* = 4. **(H,I)** HEK293A cells expressing hCB2R-GFP^2^ were treated with 1 nM–10 μM CP55940 ± 1 μM indomethacin for 10 min and ERK1/2 **(H)** or PLCβ3 **(I)** phosphorylation was measured. **(J)** HEK293A cells expressing hCB2R-GFP^2^ and βarrestin1-Rluc were treated with 1 nM–10 μM CP55940 ± 1 μM indomethacin for 30 min and BRET^2^ was measured. **(K)** HEK-CRE cells expressing hCB2R-GFP^2^ were treated with 10 μM forskolin, 1 nM–10 μM CP55940 ± 1 μM indomethacin for 1 h. Data are mean ± SEM. *N* = 4.

Indomethacin-dependent modulation of hCB1R and hCB2R signaling was examined in HEK293A cells, which are a well-established model system for studying cannabinoid receptors (Hudson et al., 2010; Laprairie et al., 2015, 2017; Tham et al., 2018). The effect of indomethacin on CP55940-dependent hCB1R and hCB2R activation was measured in HEK293A cells expressing either hCB1R-GFP^2^ or hCB2R-GFP^2^ (Figure 3 and Table 2). Indomethacin alone did not alter hCB1R-dependent βarrestin1 recruitment, ERK1/2 and PLCβ3 phosphorylation, or cAMP levels (Figures 3A,C,E,G). Indomethacin (1 μM) produced a significant leftward and upward shift in the CRCs for βarrestin1 recruitment, ERK1/2 and PLCβ3 phosphorylation, and cAMP inhibition (Figures 3A,C,E,G). Indomethacin alone did not alter hCB2R-dependent βarrestin1 recruitment, ERK1/2 or PLCβ3 phosphorylation, or cAMP inhibition in HEK293A cells expressing hCB2R (Figure 3 and Table 2). Therefore, indomethacin enhanced hCB1R-dependent signaling, and not hCB2R-dependent signaling, in a manner consistent with a PAM.

**TABLE 2:** Potency and efficacy of indomethacin at modulating agonist-dependent signaling.

	**EC_50_ (nM) (95% CI)**		***E*_max_ (%) ± SEM**	
	**CP55940**	**+1 μM Indomethacin**	**CP55940**	**+1 μM Indomethacin**
**HEK hCB1R^a^**				
ERK	340(240–480)	170 (100–270)	1005.6	1104.0
PLCβ3	350(230–520)	180 (120–280)	1005.7	1205.0^∗^
βarrestin1	240(88–570)	170 (75–730)	10012	11010
cAMP	160(83–290)	150 (65–300)	0.07.9	−8814^∗^
**HEK hCB2R^b^**				
ERK	390(210–660)	440 (230–800)	1009.2	10911
PLCβ3	500(270–870)	450 (250–770)	10010	1039.1
βarrestin1	490(310–760)	470 (290–750)	1008.3	1118.6
cAMP	350(190–600)	590 (360–960)	0.06.1	−268.5

**CHO hCB1R^c^**				
	**EC_50_ (nM) (95% CI)**		***E*_*max*_ (%) ± SEM**	
	**CP55940**	**100 nM CP55940 + Indomethacin**	**CP55940**	**100 nM CP55940 + Indomethacin**

cAMP	140(71–285)	10 (0.61–160)	0.018	2211
βarrestin2	620(240–1,600)	570 (380–850)	10011	1104.0

	**EC_50_ (nM) (95% CI)**		***E*_max_ (%) ± SEM**	
	**AEA**	**100 nM AEA + Indomethacin**	**AEA**	**100 nM AEA + Indomethacin**

cAMP	2,900(260–3,300)	1.9 (0.06–6.1)	5.93.9	235.9
βarrestin2	>10,000	>10,000	162.1	181.4

**FIGURE 4:**
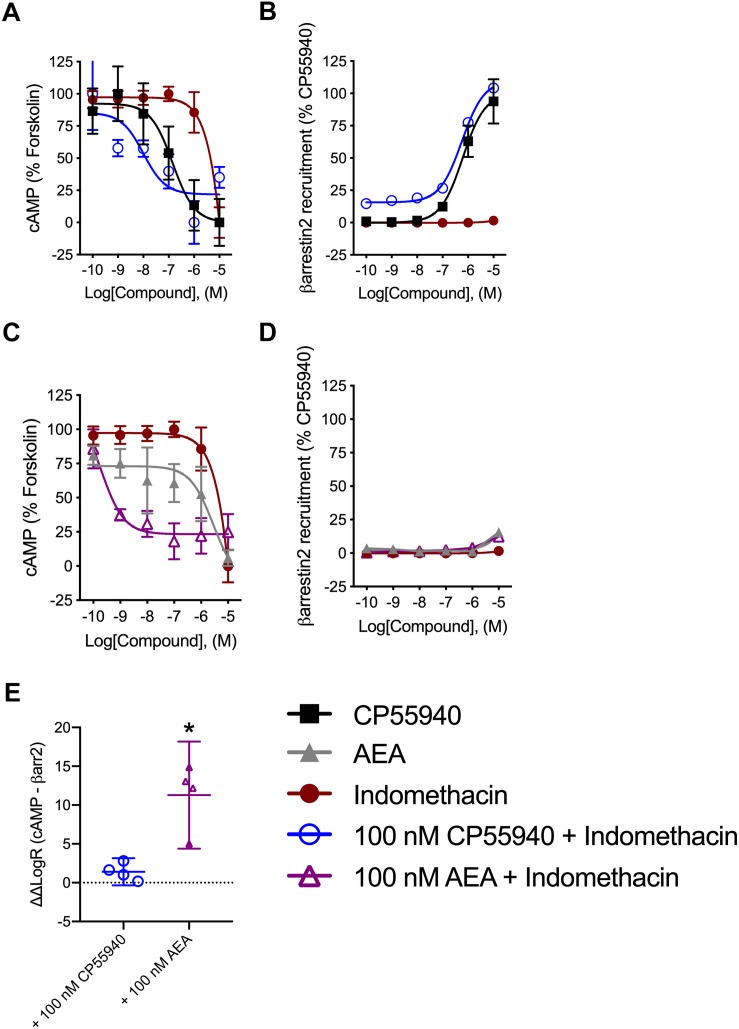
Analysis of indomethacin bias at hCB1R in CHO cells. CHO HitHunter cAMP cells **(A,C)** or PathHunter βarrestin2 cells **(B,D)** stably expressing hCB1R were treated with 0.1 nM–10 μM CP55940, AEA, indomethacin, 100 nM CP55940 + 0.1 nM–10 μM indomethacin, or 100 nM AEA + 0.1 nM = 10 μM indomethacin for 90 min. CHO HitHunter cAMP cells were also treated with 10 μM forskolin. hCB1R-depednent inhibition of forskolin-induced cAMP accumulation **(A,C)** or βarrestin2 recruitment **(B,D)** was measured. Data are mean ± SEM. *N* = 5 in panels **(A,C)**, *N* = 6 in panels **(B,D)**. **(E)** Data were fit to the operational model to calculate ΔΔLogR (cAMP–βarrestin2) such that values >0 represent bias for inhibition of cAMP and values <0 represent bias for recruitment of βarrestin2. Data are individually plotted with mean and 95% confidence interval. ^∗^*P* < 0.05 compared to 0 as determined by 95% confidence interval. *N* = 4.

*^*a*^Data are from Figures 3A,C,E,G. ^*b*^Data are from Figures 3H–K. ^*c*^Data are from Figure 4. ^∗^*P* < 0.01 compared to CP55940 as determined by one-way ANOVA followed by Tukey’ *post hoc* analysis. *N* = 4 (HEK hCB1R and HEK hCB2R), *N* = 5 (CHO hCB1R cAMP), *N* = 6 (CHO hCB1R βarrestin2).*

Indomethacin-dependent modulation of hCB1R signaling was further assessed in the DiscoveRx CHO HitHunter and PathHunter cells for βarrestin2 recruitment and cAMP inhibition in the presence of 100 nM CP55940 or AEA in order to assess ligand bias, PAM activity in the presence of the endogenous agonist, and probe dependence between CP55940 and AEA (Figure 4). Indomethacin alone did not alter hCB1R-dependent cAMP inhibition or βarrestin2 recruitment. Indomethacin enhanced 100 nM CP55940-dependent cAMP inhibition and βarrestin2 recruitment (Figures 4A,B). Further, indomethacin enhanced 100 nM AEA-dependent inhibition of cAMP but did not alter AEA-dependent βarrestin2 recruitment (Figures 4C,D). Indomethacin in the presence of CP55940 did not display bias between cAMP inhibition and βarrestin2 recruitment, whereas indomethacin in the presence of AEA did selectively enhance inhibition of cAMP relative to βarrestin2 recruitment, as determined by fitting these data with the operational model (Figure 4E). Therefore, indomethacin displayed hCB1R PAM activity with probe-dependence for AEA-dependent inhibition of cAMP.

### RT-PCR

**FIGURE 5:**
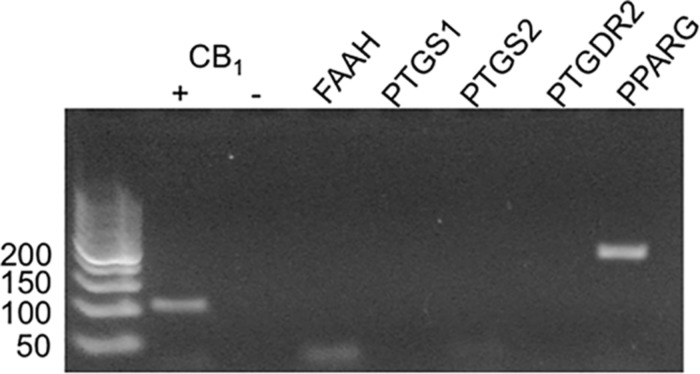
mRNA expression of potential indomethacin targets in HEK293A cells. The expression of several gene transcripts whose protein products are considered targets for indomethacin was evaluated in HEK293A cells using RT-PCR. hCB1R cDNA was detectable in cells transfected with hCB1R-GFP^2^ (+) and not untransfected cells (-). FAAH, fatty acid amide hydrolase; PTGS1 and 2, COX-1 and -2; PPARG, PPARγ.

Indomethacin is thought to interact with a number of targets, including COX-1 (*PTGS1*), COX-2 (*PTGS2*), the prostaglandin D2 receptor 2 (PTGDR2/CRTH2/PGD2; *PTGDR2*), peroxisome proliferator-activated receptor γ (PPARγ; *PPARG*), and fatty acid amide hydrolase (FAAH; *FAAH*) (Lehmann et al., 1997; Sawyer et al., 2002; Hata et al., 2005; Sugimoto et al., 2005; Holt et al., 2007). To determine whether indomethacin could have affected non-CB1R targets in HEK293A cells, mRNA was isolated, and COX-1, COX-2, PTGDR2, PPARγ, and FAAH transcripts levels were assessed by RT-PCR. hCB1R was readily detectable in HEK293A cells transfected with the hCB1R-GFP^2^ plasmid, but not detected in non-transfected HEK293A cells (-) (Figure 5). PPARγ transcript was detected, but no transcripts were detected for FAAH, COX-1, COX-2, or PTGDR2 (Figure 5). Therefore, the indomethacin-dependent enhanced CB1R signaling observed in HEK293A cells occurred via allosteric modulation of CB1R, and not through other protein targets of indomethacin. Indomethacin-mediated CB1R PAM activity may be less-evident in cell culture systems where COX-1, COX-2, PTGDR2, PPARγ, and FAAH are expressed and *in vivo*.

### *In vivo* Analyses

**FIGURE 6:**
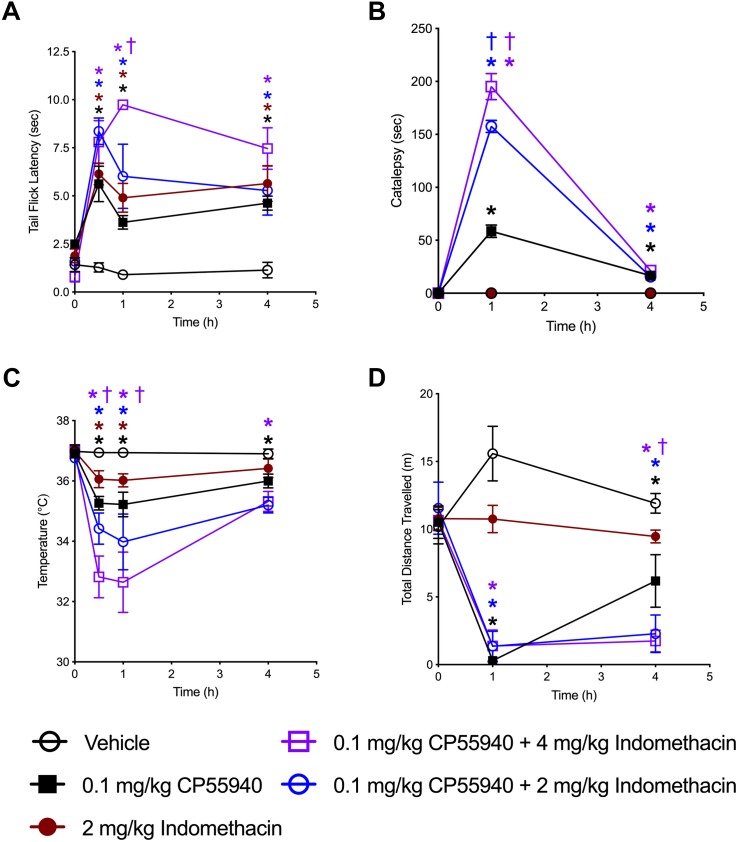
Indomethacin enhanced CP55940-dependent tetrad effects. Seven-week old, male, C57BL/6 mice were injected (*i.p.*) with vehicle, CP55940 (0.1 mg/kg), indomethacin (2 mg/kg), CP55940 (0.1 mg/kg) + indomethacin (2 mg/kg), or CP55940 (0.1 mg/kg) + indomethacin (4 mg/kg) and tetrad tests were completed as follows: tail flick latency at 0 (prior to treatment), 0.5, 1, and 4 h after injection **(A)**, catalepsy at 0, 1, and 4 h after injection **(B)**, internal body temperature at 0, 0.5, 1, and 4 h after injection **(C)**, total distance traveled in the open field at 0, 1, and 4 h after injection **(D)**. ^∗^*P* < 0.01 compared to vehicle within time point, ^†^*P* < 0.01 compared to CP55940 alone within timepoint, as determined via two-way ANOVA followed by Bonferroni’s *post hoc* analysis. Data are mean ± SEM. *N* = 5 per treatment group.

The ability of indomethacin to enhance CB1R-dependent effects was assessed *in vivo* using tetrad analysis over 4 h (indomethacin *t*_1__/__2_ in mouse 51 min, 4.7 half-lives) (Remmel et al., 2004). Tail flick latency was increased by both CP55940 (0.1 mg/kg) and indomethacin (2 mg/kg) at 0.5, 1, and 4 h compared to vehicle treatment, and increased by the combination of CP55940 and indomethacin (4 mg/kg) at 1 h compared to CP55940 or indomethacin alone (Figure 6A). Catalepsy was increased by CP55940 alone at 1 and 4 h, but not indomethacin (Figure 6B). Catalepsy time was significantly increased by 2 and 4 mg/kg of indomethacin with CP55940 compared to CP55940 alone at 1 h (Figure 6B). Body temperature was reduced by both CP55940 and indomethacin at 0.5 and 1 h compared to vehicle treatment, and further reduced by the combination of CP55940 and indomethacin (4 mg/kg) at 0.5 and 1 h compared to CP55940 or indomethacin alone (Figure 6C). Locomotion (i.e., distance traveled in the open field) was reduced by CP55940 at 1 and 4 h compared to vehicle treatment, and further reduced by the combination of CP55940 and indomethacin (4 mg/kg) at 4 h compared to CP55940 or indomethacin alone (Figure 6D).

### *In silico* Ligand Docking

**FIGURE 7:**
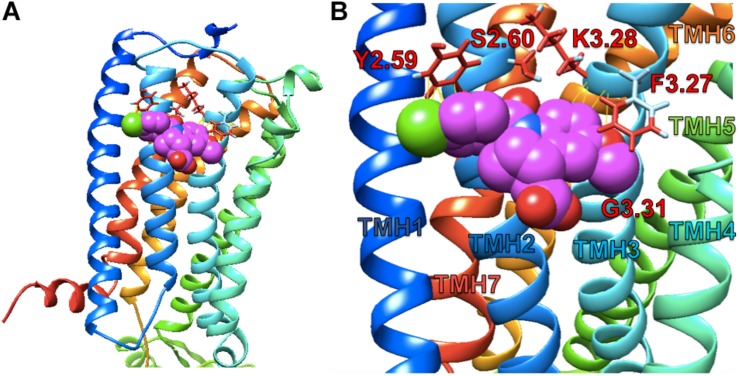
Indomethacin docking to CB1R 5XRA (agonist-bound). **(A)** The perspective is from the lipid bilayer. Helices are blue (I), light blue (II), turquoise (III), seafoam (IV), green (V), gold (VI), and orange (VII). Indomethacin is shown in magenta. **(B)** Image as in **(A)** at a closer perspective. Interacting amino acid residues are named according to the Ballesteros and Weinstein (1995) system. Transmembrane helices (TMH) are labelled by number.

Simulated docking of indomethacin to CB1R-5XRA was modeled in AutoDock 4.2.6. to predict possible binding sites of indomethacin in an active conformation of CB1R bound orthosteric agonist AM11542 (a CP55940 derivative) (Figure 7). Indomethacin bound a subset of residues on the exterior surface of transmembrane helices 2 and 3 (Figure 7) that do not overlap with those of the orthosteric agonist (S1.39, F2.57, F2.61, F2.64, H2.65, F3.25, L3.29, V3.32, F3.36, L5.40, W5.43, M6.55, W6.48, L6.51, F7.35, A7.36, S7.39, M7.40, C7.42, and L7.43) (Hua et al., 2017). Amino acid residue K3.28 has been previously reported to interact with Org27569 and PSNCBAM-1 (Hurst et al., 2006). Importantly, amino acid residues Y2.59, F3.27 were recently reported to interact with the well-known CB1R PAM GAT229 and also interacted with indomethacin in this model (Hurst et al., 2019), supporting a shared binding site for these CB1R PAM. Ligand affinity was estimated for the 5XRA-CB1R model in AutoDock 4.2.6. for indomethacin and the estimated *K*_A_ value for indomethacin was 450 nM, which is similar to the potency observed for indomethacin as a CB1R PAM *in vitro*.

## Discussion

In this study, we present evidence that the NSAID indomethacin acted as a PAM of CB1R *in vitro* and *in vivo*. Indomethacin is known to interact with a number of proteins, including the multidrug resistance proteins 1 and 4, COX-1, COX-2, PTGDR2/CTRH2, PPARγ, and the AEA-metabolizing enzyme FAAH (Lehmann et al., 1997; Hata et al., 2005; Sugimoto et al., 2005; Holt et al., 2007). The non-selective activity of indomethacin may explain several of the side effects associated with this drug, including dyspepsia, heartburn, diarrhea, edema, and hypertension (Fowler, 1987). In the present study, the CB1R PAM activity of indomethacin ranged in potency from 10 nM (cAMP inhibition assay) to 570 nM (βarrestin2 recruitment assay) in the presence of CP55940 (Table 2). By comparison, indomethacin inhibits COX-1 (250 nM), PTGDR2/CTRH2 (20–790 nM), and PPARγ (40 nM) within a similar concentration range to the potencies observed for CB1R-dependent signaling (Lehmann et al., 1997; Sawyer et al., 2002; Hata et al., 2005; Sugimoto et al., 2005). In contrast to these effects, indomethacin has been shown to inhibit MRP1 and 4 (11 and 102 μM, respectively), FAAH (1.2 μM), and COX-2 (2.5 μM) at much higher concentrations (Reid et al., 2003; Holt et al., 2007). Several additional CNS-specific side effects associated with indomethacin use but not other NSAIDs, such as headache, vertigo, and dizziness, blurred vision, and psychosis following prolonged use, may be explained by the drug’s modulation of the endocannabinoid system and/or CB1R (Wiley et al., 2006; Parvathy and Masocha, 2015). The endogenous substrates of COX-1, COX-2, PPARγ, FAAH, and CB1R share similar chemical structures and physical properties. Moreover, exogenous cannabinoids such as Δ^9^-tetrahydrocannabinol (THC) are known to modulate COX enzymes (Chen et al., 2013). The CB1R PAM activity of indomethacin – and similar observations such as CB1R PAM activity by fenofibrate (PPARγ agonist) (Priestley et al., 2015), and FAAH inhibition by acetaminophen (Ottani et al., 2006) – indicate a pharmacological overlap between these proteins.

*In vitro*, indomethacin enhanced CP55940 binding and activation of hCB1R in [^35^S]GTPγS, ERK1/2, PLCβ3, βarrestin1, βarrestin2, and cAMP assays. Indomethacin also enhanced AEA-dependent inhibition of cAMP – but did not enhance AEA-dependent βarrestin2 recruitment – indicating indomethacin’s effects are probe-dependent, biased toward cAMP inhibition in the presence of endogenous agonist, and occur in the presence of endogenous agonist. These experiments were conducted in acute treatment paradigms and in cell signaling systems that overexpress human CB1R. Subsequent studies exploring indomethacin-dependent modulation of CB1R in long-term treatment, endogenous expression systems, and on electrophysiological outputs will enhance our understanding of indomethacin PAM activity (Straiker et al., 2018). Binding of indomethacin to an allosteric site of CB1R could have shifted the equilibrium of CB1R from the inactive R state, to the more active R^∗^ state (Iliff et al., 2011; Fay and Farrens, 2012; Shore et al., 2014). Our *in silico* modeling of CB1R with the active R^∗^ state model (5XRA) further supports indomethacin binding a unique allosteric pocket distinct from Org27569 or PSNCBAM-1 (Iliff et al., 2011; Fay and Farrens, 2012). The CB1R allosteric modulators Org27569 and PSNCBAM-1 have been shown to promote R^∗^ state conformation and increase orthosteric ligand binding (Iliff et al., 2011; Fay and Farrens, 2012; Shore et al., 2014); and our modeled indomethacin binding site overlaps that of the recently modeled GAT229 CB1R PAM binding site (Hurst et al., 2019). Org27569 and PSNCBAM-1 enhance CP55940 binding, but not CB1R-dependent signaling (Price et al., 2005; Shore et al., 2014), whereas indomethacin enhanced both binding and signaling because of its topologically distinct binding site.

*In vivo*, indomethacin was able to promote anti-nociceptive and hypothermic effects alone at 2 mg/kg and enhance all 4 CP55940-dependent tetrad effects at 2 and 4 mg/kg. Indomethacin may have induced tetrad effects alone via inhibition of its other known targets, COX-1/2 and FAAH, which would lead to elevated levels of endocannabinoids. The potentiating effects of indomethacin ceased within the 4 h time course of the experiment, which is consistent with the 51 min half-life of indomethacin in mice (Remmel et al., 2004). Moreover, although 90% of indomethacin is plasma-protein bound, free [^14^C]indomethacin has been shown to rapidly penetrate the rat brain via transporter-independent mechanisms (Parepally et al., 2006). These data support the hypothesis that *in vivo* effects observed in our study were brain CB1R-dependent. Other CB1R PAMs that contain indole-2-carboxamides, such as GAT211 and ZCZ011, enhance some CB1R-dependent effects *in vivo* (Slivicki et al., 2018). Other CB1R allosteric ligands, such as Org27569 and PSNCBAM-1, have limited efficacy *in vivo*, potentially because of poor pharmacokinetic properties (Ignatowska-Jankowska et al., 2015; Gamage et al., 2017).

Wiley et al. (2006) reported that indomethacin (10 or 30 mg/kg) enhanced AEA-dependent (30 mg/kg) hypolocomotion, anti-nociception, hypothermia, and immobility in ICR mice. The authors suggest that indomethacin may have potentiated AEA’s effects via reduced metabolism of AEA (Wiley et al., 2006), which is supported by other studies (Fowler et al., 1997a, b, 1999; Holt et al., 2007). Parvathy and Masocha (2015) have also reported that indomethacin reduces neuropathic thermal paclitaxel-induced hyperalgesia via CB1R. Our studies utilized a lower dose of indomethacin (2 or 4 mg/kg) in an acute treatment paradigm and demonstrated the potentiation of CP55940-dependent effects. Indomethacin, and other COX inhibitors, have also been shown to reduce the efficacy of chronically administered CB1R agonists *in vivo* (Yamamguchi et al., 2001; Anikwue et al., 2002). Previous studies that described interactions between COX inhibitors and CB1R agonists utilized chronically administered cannabinoid agonist. Here, the acute co-administration of CP55940 and indomethacin enhanced by CP55940-mediated effects (Yamamguchi et al., 2001; Anikwue et al., 2002). Although we did not explore the possible role of metabolites in our acute study, it is possible that the metabolites of indomethacin may also affect the activity of CB1R and other targets in acute and chronic treatment paradigms. Chronic cannabinoid administration is known to produce receptor desensitization and downregulation, which may account for the decreased efficacy observed in earlier studies. Future studies will explore chronic CB1R-dependent effects *in vivo*.

Indomethacin enhanced the efficacy, potency, and ligand binding of CB1R agonists *in vitro* and *in vivo* in a manner consistent with positive allosteric modulation. Therefore, indomethacin may be a useful probe compound to understand the structure-activity relationship of CB1R allosteric modulators, and modulators of FAAH and COX enzymes, and in the development of novel therapeutic compounds with specificity for these components of the endocannabinoid system.

## Data Availability Statement

The raw data supporting the conclusions of this manuscript will be made available by the authors, without undue reservation, to any qualified researcher.

## Ethics Statement

The animal study was reviewed and approved by the Dalhousie University Animal Care Committee.

## Author Contributions

RL designed, executed, and analyzed the experiments, and contributed to the writing and editing of the manuscript. KM, AZ, and LS designed, executed, and analyzed the experiments. MK, RP, and ED-W designed the experiments, and contributed to the writing and editing of the manuscript. GT proposed the hypothesis that indomethacin can act as a CB1R PAM, provided the research material, analyzed the experiments, and contributed to the writing and editing of the manuscript.

## Conflict of Interest

The authors declare that the research was conducted in the absence of any commercial or financial relationships that could be construed as a potential conflict of interest.
