# Supplementary material for: Indomethacin Enhances Type 1 Cannabinoid Receptor Signaling
Source: Front Mol Neurosci. 2019 Oct 18;12:257. doi: 10.3389/fnmol.2019.00257 (PMC6813218; doi:10.3389/fnmol.2019.00257)
Supplement: Supplementary file 1 [file Table_1.docx]

| **Supplementary Table 1. PCR primers used in this study.** | | | | |
| --- | --- | --- | --- | --- |
| **Target** | **Oligonucleotide Sequence (5' - 3')** | **Anneal. Temp. (°C)** | **MgCl_2_ (mM)** | **Product length (bp)** |
| CB1R | GGGCAAATTTCCTTGTAGCA | 58 | 1 | 129 |
|  | GGCTAACGTGACTGAGAAA |  |  |  |
| FAAH | AAGGTGATTTCGTGGACCCC | 58 | 4 | 150 |
|  | TTCCAGCCGAACGAGACTTC |  |  |  |
| PTGS1 | TCTTGCTGTTCCTGCTCCTG | 58 | 2 | 146 |
|  | GTCACACTGGTAGCGGTCAA |  |  |  |
| PTGS2 | GCCAAGCACTTTTGGTGGAG | 57 | 3 | 356 |
|  | CCTTTCTCCGCAACAGGAGT |  |  |  |
| PTGDR2 | GCAACCTCTATGCGATGCAC | 57 | 2 | 279 |
|  | AGCGCGATACTTGGGAGAAA |  |  |  |
| PPARG | TCAGAAACGGGGAGTAACCG | 57 | 2 | 262 |
|  | ACGGAGCTGATCCCAAAGTT |  |  |  |
